# From Site Data to Safety Assessment: Analysis of Present and Future Hydrological Conditions at a Coastal Site in Sweden

**DOI:** 10.1007/s13280-013-0394-6

**Published:** 2013-04-26

**Authors:** Sten Berglund, Emma Bosson, Mona Sassner

**Affiliations:** 1 HydroResearch AB, Stora Marknadsvägen 15S (12th Floor), 183 34 Täby, Sweden; 2 Swedish Nuclear Fuel and Waste Management Co (SKB), Box 250, 101 24 Stockholm, Sweden; 3 DHI Sverige AB, Svartmangatan 18, 111 29 Stockholm, Sweden

**Keywords:** Hydrology, Hydrological modeling, Landscape development, Biosphere, Forsmark

## Abstract

This paper presents an analysis of present and future hydrological conditions at the Forsmark site in Sweden, which has been proposed as the site for a geological repository for spent nuclear fuel. Forsmark is a coastal site that changes in response to shoreline displacement. In the considered time frame (until year 10 000 ad), the hydrological system will be affected by landscape succession associated with shoreline displacement and changes in vegetation, regolith stratigraphy, and climate. Based on extensive site investigations and modeling of present hydrological conditions, the effects of different processes on future site hydrology are quantified. As expected, shoreline displacement has a strong effect on local hydrology (e.g., groundwater flow) in areas that change from sea to land. The comparison between present and future land areas emphasizes the importance of climate variables relative to other factors for main hydrological features such as water balances.

**Electronic supplementary material:**

The online version of this article (doi:10.1007/s13280-013-0394-6) contains supplementary material, which is available to authorized users.

## Introduction

Analyses of hydrological processes such as groundwater and surface water flow and water exchanges between atmosphere, regolith, and vegetation are often fundamental components in environmental assessments and geoscientific research studies. Applications may be focused on the water itself (e.g., for drinking water supply), or consider cases where water primarily is of interest as a carrier of chemical substances (e.g., in contaminant transport) or as an environment where biogeochemical processes take place. In applications like these, hydrological models are often developed and used to predict or otherwise describe the future. The time periods considered in most of these studies of future hydrology are relatively short, on the order of a few decades up to, say, one hundred years. However, in recent years there has been an increasing interest in issues involving much longer time scales, such as long-term climate change and risk assessments of long-lived contaminants. When considering these longer time perspectives, also changes in the studied systems as such must be taken into account; for example, Rowland et al. (2010) emphasize the need for integrating hydrological, ecological, geomorphological, and geochemical processes in studies of changes in permafrost systems.

This paper presents modeling of present and future hydrological conditions at the Forsmark site in Sweden. In 2009, Forsmark was selected by the Swedish Nuclear Fuel and Waste Management Company (SKB) as the intended site for the planned geological repository for spent fuel from the Swedish nuclear power plants, and an application for constructing and operating the repository was submitted to the authorities in 2011 (Kautsky et al. 2013). As a basis for site selection and the application, multi-disciplinary site investigations of bedrock, regolith, and surface ecosystems were performed by SKB. Site descriptive models (SKB 2008) focusing on the present state of the site were developed and then used as a starting point for repository design, environmental impact assessment, and the assessment of long-term radiological safety (SKB 2011), which were all important parts of the submitted application.

Quantification of hydrological processes was a central component in many parts of the safety assessment of the planned repository, including the analysis of the conditions within the repository tunnels and deposition holes, the quantification of radionuclide transport from the repository to the biosphere, and the calculations of radiation doses in the biosphere. Therefore, a set of different hydrological/hydrogeological models, focusing on different processes, issues, or parts of the hydrological system, were developed during the site investigations and the safety assessment of the spent fuel repository. Specifically, surface hydrology/near-surface hydrogeology models (Johansson 2008; Bosson et al. 2008, 2010) were developed for the surface system/biosphere description, and bedrock hydrogeology models (Follin 2008; Selroos and Follin 2010) were developed for the geosphere/repository description. Meteorological and hydrological data from the site investigations at Forsmark have been used also in a number of scientific studies addressing hydrological and solute transport issues not directly related to nuclear waste (e.g., Jarsjö et al. 2008; Juston et al. 2009; Destouni et al. 2010). These studies have contributed to the site understanding and provided support to the conceptual and numerical modeling performed by SKB. Recently, modeling studies of future hydrological conditions at Forsmark have been reported by Bosson et al. (2012a, b).

The present study utilizes, extends, and analyses simulation results reported by Bosson et al. (2008, 2010). Compared with most other hydrological studies, it considers a relatively long time period, from today until 10 000 ad. This means that some long-term processes that usually can be ignored in model development are potentially of importance in the hydrological modeling. In particular, the Forsmark site is situated on the Baltic Sea coast at a location where shoreline displacement takes place at a rate implying that this process needs to be taken into account within the time frame under consideration (Lindborg 2010; Lindborg et al. 2013). Available shoreline displacement modeling results (see SKB 2010 for a discussion) show that new land areas will be created outside the present coastline during the considered time period. Lakes and streams are formed in depressions in the former sea bottom and will undergo the same succession, from lakes to wetlands and possibly further to agricultural areas or forests, as can be observed in the present land areas (Lindborg et al. 2013). In the model development presented here, future shoreline locations and landscape succession are considered, together with associated changes in regolith distribution and stratigraphy (due to processes such as wave action and sedimentation), climate, and vegetation.

A main objective of this study is to present a methodology for site-specific model development and application of model results in safety assessment calculations. We also wish to analyze further some aspects of the modeling believed to be of particular importance and general interest. Specifically, the model calibration based on measured and modeled data for present conditions, the development of models for future hydrological conditions, and the application of hydrological model results in the compartment model used in radionuclide transport and radiation dose calculations are discussed in the paper.

## Methods and Models

### Site Overview

**Fig. 1:**
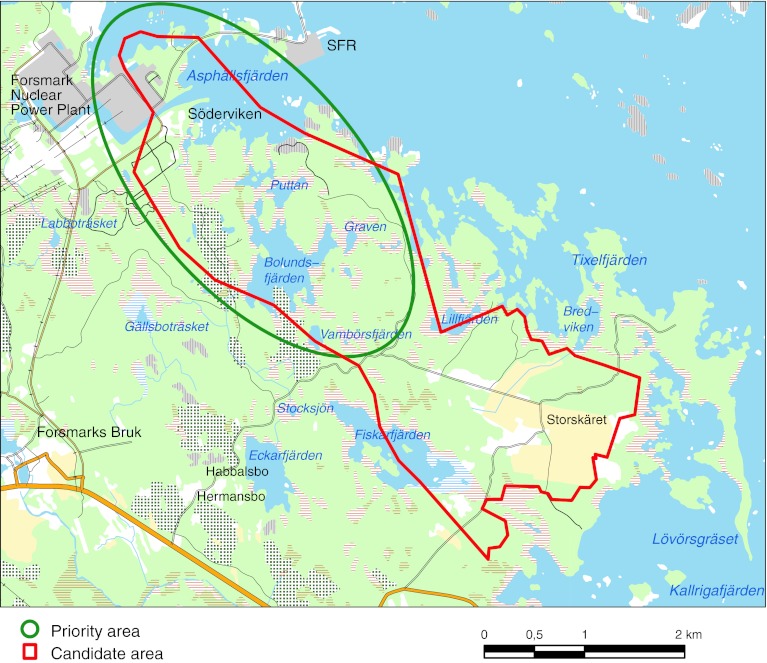
Candidate and priority areas (see text) considered in the Forsmark site investigations and modeling for the planned spent fuel repository. The main lakes (e.g., Lake Bolundsfjärden) and sea bays (e.g., Asphällsfjärden) are also indicated. The *gray-colored area* in the *upper left corner* is the Forsmark nuclear power plant and ‘SFR’ indicates the existing repository for short-lived radioactive waste

Forsmark is located in central Sweden in the municipality of Östhammar, approximately 120 km north of Stockholm (cf. SKB 2008). The area is situated on the coastline of Öregrundsgrepen, a funnel-shaped bay of the Baltic Sea, and is characterized by small-scale topography at low altitude. Figure 1 shows some of the most important hydrological objects in the investigated area. It also indicates the extent of the ‘candidate area’, where most of the site investigations were made, and the ‘priority area’, where the repository is planned to be built and on which the later stages of the investigations therefore were focused. The whole area is located below the highest coastline associated with the last glaciation, and large parts of the candidate area emerged from the Baltic Sea only during the last 2000 years. Forest is the dominant land cover and granitic rocks dominate the bedrock of the area (SKB 2008).

Post-glacial land uplift, in combination with a flat topography and the still on-going shore-level regression of ca. 6 mm per year, implies fast shoreline displacement in which sea bottoms are continuously transformed into new terrestrial areas or freshwater lakes. This has resulted in a very young terrestrial system containing a number of recently formed shallow lakes and wetlands. There are four main lakes in the investigated area, Lake Fiskarfjärden, Lake Bolundsfjärden, Lake Eckarfjärden, and Lake Gällsboträsket (Fig. 1), which all are smaller than 1 km^2^ and very shallow. No major water courses run through the central parts of the site investigation area. Wetlands are frequent in this young landscape.

**Fig. 2:**
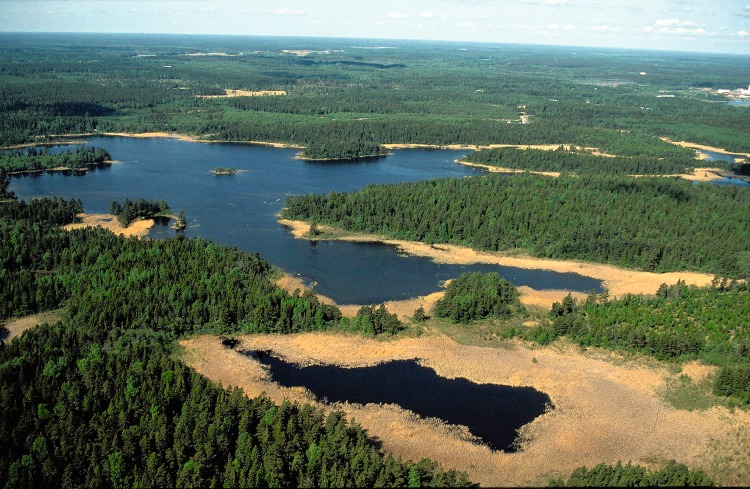
Lakes with surrounding reed-covered mires in Forsmark illustrating typical areas considered as biosphere objects in the safety assessment modeling

The regolith consists of unconsolidated Quaternary deposits overlying the bedrock. It is shallow, usually less than 5 m deep, and consists mostly of till, except in lake and wetland areas where glacial clay and post-glacial clays and gyttja are also found. Discharge areas for groundwater passing through the planned repository volume at ca. 500 m depth are of particular interest for the safety assessment. Groundwater discharge takes place in depressions in the surface topography, which in Forsmark typically contain lakes surrounded by reed-covered wetlands. Figure 2 illustrates typical potential discharge areas in Forsmark. A description of the identified discharge areas associated with the Forsmark repository is given by Berglund et al. (2013).

### Hydrological Investigations and Site Description

**Fig. 3:**
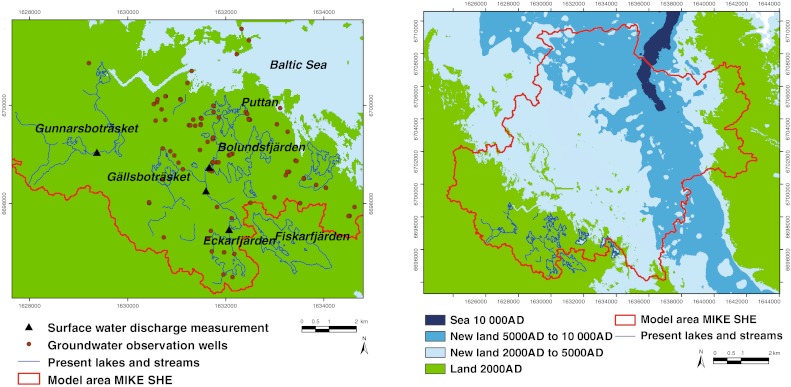
Measurement stations used in model calibration (*left*), and present and future land and sea areas considered in the hydrological modeling (*right*). Displayed ‘Groundwater observation wells’ include both boreholes in bedrock and monitoring wells (standpipes) in regolith; note that not all boreholes and standpipes in the investigation area are shown in the figure. The map to the *right* illustrates shoreline displacement; in 10 000 ad the sea is limited to a narrow bay (the *dark blue area*)

The installations and investigations providing meteorological and hydrological data from the Forsmark site included establishment and operation of two local weather stations, four discharge measurement stations in streams, six water-level measurement stations in lakes and two in the Baltic Sea, and a large number of boreholes in regolith and bedrock [Fig. 3; see Johansson (2008) for details]. The meteorological measurements provided time-series data from the candidate area shown in Fig. 1 on precipitation, air temperature and humidity, barometric pressure, wind speed and direction, and global radiation. The site-specific potential evapotranspiration, which was an important input to the hydrological modeling, was calculated based on these data. Drillings in the regolith resulted in more than 90 installations for measurements of groundwater levels and/or hydrochemical sampling. Automatic measurements of high-resolution groundwater level time series were performed in ca. 50 groundwater monitoring wells. Hydraulic tests for determining the hydraulic conductivities of the various regolith materials (slug tests in till in most cases) were carried out at about 70 locations. Automatic data collection was also performed at all surface water-level and discharge stations.

The site investigations show that the hydrology in Forsmark is characterized by a number of relatively small and shallow lakes, frequent wetland areas, and small streams where flow may cease during dry summers. A long-term overall water balance, including a precipitation of 560 mm/year, an actual evapotranspiration of 400 mm/year and a runoff of 160 mm/year, was estimated based on 30-year precipitation data from surrounding areas and the relatively short-term site-specific meteorological and hydrological monitoring data (Johansson 2008).

The groundwater table in the regolith is very shallow, and is closely correlated to the topography of the ground surface. This implies that there is a strong interaction between evapotranspiration, soil moisture, and groundwater. Diurnal fluctuations of groundwater levels, driven by evapotranspiration cycles, are evident in the data from many of the groundwater monitoring wells in the regolith. Direct recharge from precipitation is the dominant source of groundwater recharge. However, groundwater level measurements performed near Lake Bolundsfjärden and Lake Eckarfjärden (Fig. 1) show that the lakes may act as recharge sources to the till aquifers in the immediate vicinity of the lakes under dry summer conditions.

The small-scale topography and the decreasing hydraulic conductivity with depth in the till imply that many small catchments are formed with local shallow groundwater flow systems in the regolith, and that the majority of the groundwater moves along these shallow flow paths. The local, small-scale recharge and discharge areas involving groundwater flow systems restricted to the regolith overlie larger-scale flow systems associated with groundwater flow at greater depths. The groundwater level in the upper bedrock is flat and shows no such strong coupling to the surface topography. This is most evident in the priority area (Fig. 1) where the upper part (ca. 150 m) of the bedrock is known to have frequent horizontal and nearly horizontal fractures of high transmissivity.

The measured lake water level-groundwater level relationships indicate that the lake sediments, the underlying till, and/or the uppermost bedrock have low vertical hydraulic conductivities. If the surface water–groundwater hydraulic contact had been good, the observed situation with groundwater level drawdown from evapotranspiration and pumping tests extending below the lakes would not exist. The flow systems around and below the lakes appear to be quite complex, with differences in groundwater flow directions and groundwater–surface water interactions between different parts of the lakes and between different times of the year.

### Numerical Modeling of Present Conditions and Calibration Methodology

The numerical modeling of surface hydrology and near-surface hydrogeology in Forsmark was performed using the MIKE SHE modeling tool (Graham and Butts 2005; Butts and Graham 2008). MIKE SHE enables dynamic, physically based modeling of all main processes in the land phase of the hydrological cycle, including saturated and unsaturated groundwater flow, surface water flow, water uptake in vegetation, and evapotranspiration processes. For the modeling of surface water flow in streams, MIKE SHE is fully integrated with a channel-flow code (MIKE 11), such that the exchange of water between streams and the surrounding groundwater can take place continuously during the simulation. MIKE SHE can also be used for performing transport simulations (see Bosson et al. 2010, 2012b; Berglund et al. 2013).

The modeling focusing on the present conditions used time series input data from the site investigation period. Specifically, model calibration and testing were performed using site data from the period May 2003 to March 2007, with a MIKE SHE model covering 37 km^2^ having a horizontal grid resolution of 40 m (Bosson et al. 2008, 2012a). The calibration attempted to match measured and calculated time series, with a temporal resolution of 1 day, using field data on surface water levels and discharges (from five and four stations, respectively), groundwater levels in regolith (34 monitoring wells), and groundwater levels in bedrock (39 borehole sections in 19 boreholes). The calibration procedure first focused on the surface water system and the overall water balance, then shifted to the groundwater levels in the regolith, and finally considered the groundwater head elevations in the bedrock (Bosson et al. 2008). Calibration was made in two main stages, where the second stage was a recalibration initiated partly due the arrival of new input data (an updated regolith model) and partly in an attempt to improve some specific aspects of the model performance. In total, 36 model simulations were performed during the calibration process.

Model performance was quantified by using the mean error (ME), i.e., the mean difference between the daily values in the measured and modeled time series (could be positive or negative) for a particular measurement station, and the mean absolute error, MAE, which is the mean of the absolute differences (always positive) between the time series. The calibration targets for the groundwater level in the regolith were to obtain average ME and MAE of less than 0.20 and 0.40 m, respectively, for the groundwater monitoring wells. For cumulative surface water discharges, the calibration target was to be below a maximum difference of 15 % between measured and modeled water volumes at the end of the considered time period. Finally, another important criterion was to keep the hydraulic variables within physically realistic ranges, not diverging from site observations.

### Modeling Future Hydrological Conditions

Hydrological models describing future conditions at the site were developed to produce parameter values (hydrological fluxes) for the calculations of radionuclide transport and associated radiation doses to be carried out within the biosphere modeling in the safety assessment. Other purposes of the numerical modeling were to investigate possible future surface water and groundwater conditions, and to what extent lakes, wetlands, and other objects of interest might differ from those observed at the site today. The modeling of future site hydrology used the description of the present state as a basic input. The other main input was the assessment and quantitative modeling of the various processes driving the succession of the regolith and hydrological objects at the site.

A description of landscape development at Forsmark is provided by Lindborg et al. (2013). The main processes affecting long-term site development are the partly related processes of shoreline displacement and climate change (see SKB 2010; Näslund et al. 2013). In this work, models intended to represent possible hydrological conditions at 5000 ad and 10 000 ad were produced; the modeled shore levels are −15.0 m at 5000 ad and −31.4 m at 10 000 ad (expressed in the Swedish RHB70 elevation system). Figure 3 shows how the area changes from present conditions (green areas are land) to the situation at 10 000 ad (only the small dark blue area is sea). The hydrological modeling considered fixed geometrical and geological conditions at these times, i.e., ‘snap shots’ in the dynamic development of the site. This means that surface hydrology was not modeled continuously in the changing landscape, in contrast to some of the dynamic processes determining, for example, the geometrical and geological conditions (Brydsten and Strömgren 2010).

The results from quantitative modeling of the succession of regolith, wetlands, and lakes presented by Brydsten and Strömgren (2010) were used in the hydrological modeling. Specifically, the changes in the upper regolith layers due to erosion and sedimentation, which caused changes in the spatial distribution and stratigraphy of regolith materials, and the terrestrialization of lakes were included in the hydrological models. In particular, lake terrestrialization (which implies that a given lake exists only during a certain period of time depending on its depth, size, and other properties) is a central process in the description of the changing site (Lindborg et al. 2013). Identification of streams in new land areas (Bosson et al. 2010) and descriptions of future vegetation and land use (Löfgren 2010) were other important components of the modeled landscape development.

The MIKE SHE model area used in the safety assessment modeling of the future Forsmark is also shown in Fig. 3. For the model to include the new land areas emerging as a result of shoreline displacement, the size of the model area was increased to 180 km^2^ (the previous model was 37 km^2^). In order to keep simulation times relatively short, the horizontal grid resolution was changed from 40 m to 80 m. As a reference, and to enable comparisons with the previous site descriptive modeling and an evaluation of the effects of the coarser resolution, the present situation was modeled also with the enlarged hydrological model.

Different combinations of shoreline locations (corresponding to different times and proportion of land and sea), regolith descriptions (present and future conditions), and climate conditions were considered, in order to evaluate potential effects of different changes of the system. Three different climate states were simulated: (i) the present temperate climate, (ii) a future warm and wet temperate climate, and (iii) a future periglacial climate with permafrost. Input data to the description of the present climate were taken from the site investigation, whereas climate modeling presented by Kjellström et al. (2009) provided input data on the future climates. For all climate cases, meteorological and hydrological data with a resolution of 1 day for a 1-year period were used as input data. In the flow simulations, the 1-year period was repeated until stable conditions were reached (i.e., stable, but varying during the year), and the last year was used to generate the desired output. A more detailed description of the handling of different climates and landscape development is given by Bosson et al. (2010, 2012b).

Results from the MIKE SHE modeling in terms of water fluxes from bedrock to regolith and between different parts of the surface system were required as input data to the radionuclide transport modeling. The radionuclide transport and resulting radiation doses were to be calculated using a compartment model that uses a simplified representation of the landscape objects, typically consisting of a lake surrounded by a mire, where modeling indicated that discharge of potentially radionuclide-bearing groundwater could occur (Lindborg 2010; Berglund et al. 2013). These objects, in the modeling referred to as ‘biosphere objects’, were subdivided in the radionuclide model into compartments representing different parts of the lake and mire areas, e.g., the water in the lake and different geological layers (Avila et al. 2010, 2013). A description of how the hydrological input to the radionuclide transport model was generated is provided in the Appendix (Electronic Supplementary Material), where also some example results are given.

## Results

### Description of Present Hydrology

Descriptive modeling of present site hydrology entailed extensive comparisons between numerical modeling results and measured data, using a relatively detailed model. This modeling also served as a reference in the safety assessment modeling otherwise focused on future site conditions where a larger model of lower spatial resolution was employed. In the following, some results of the model calibration and the comparison between model results for present conditions are presented; the site description and safety assessment models are referred to as SDM and SA models, respectively.

The calculated accumulated discharges for the four surface water discharge stations (Fig. 3) show differences between measured and calculated volumes that range between 8 and 13 % for the SDM model, whereas the corresponding range for the SA model is between 3 and 10 %. This indicates that the surface water discharge was better captured in the SA model, and hence no negative effect of the increased cell size in the numerical model was observed for this particular model output. The most downstream discharge station located in the inlet to Lake Bolundsfjärden (Fig. 3) had a difference of 3 % between measurements and model results in the SA model. The measured and modeled cumulative discharges for this station are illustrated in Fig. S3 (in Electronic Supplementary Material).

The mean MAE values for all the groundwater monitoring wells in regolith that were included in the calibration process were 0.31 m for the SA model and 0.26 m for the SDM model, whereas the mean ME values were 0.00 m and −0.04 m for the SA and SDM models, respectively. The differences between measured and observed groundwater head elevations in the bedrock were larger in both models. The mean MAE in bedrock was 0.70 m and the ME −0.69 m in the SA model, and the corresponding values were 0.68 m (MAE) and −0.66 m (ME) in the SDM model. A hydraulic pumping test performed at the site during the summer 2006 (see Follin 2008) was also simulated, and the head drawdown showed acceptable agreement with observed values in both models. It was concluded that the larger SA model reproduced the present hydrology sufficiently well, and hence could be used as a starting point for modeling future hydrology. All results presented in the following were obtained using the SA model.

The groundwater level observations within the catchment show that approximately 80 % of the observations (daily average groundwater depth) is within 1 m of the ground surface and thus have a strong correlation to surface topography (Johansson 2008). The cumulative frequency of the simulated groundwater depths for the whole land part of the MIKE SHE model area and the corresponding measured groundwater depths in the observation points within the Forsmark catchment have been calculated and compared (Fig. S3 in Electronic Supplementary Material). The model results for present conditions indicate that 80 % of the model area has groundwater depths within 1.5 m of the ground surface (cf. 20 % cumulative frequency), as compared to 1 m for the same fraction in the field observations. This difference can be explained by the fact that the model area covers a larger range in ground surface elevation than the area represented by the groundwater monitoring well locations. This means that the model area includes a larger fraction of higher-elevation areas, where the depth to the groundwater table is larger.

The water balance of the present Forsmark area has been calculated for the part of the model area constituting land today. Approximately 30 % of the precipitation leaves the model volume as runoff and the evapotranspiration constitutes approximately 70 % of the precipitated water. The water balance calculation for a selected reference year with a precipitation (P) of 583 mm and a potential evapotranspiration (PET) of 420 mm resulted in a runoff (R) of 175 mm and an evapotranspiration of 405 mm (Table S2 in Electronic Supplementary Material). This is in good agreement with the long-term annual water balance estimated using local site data and regional meteorological observations, where the runoff is 160 mm and the evapotranspiration is 400 mm for a precipitation slightly lower than that characterizing the modeled reference year (cf. above).

### Future Hydrological Conditions

The modeling of future hydrological conditions at Forsmark considered shorelines corresponding to the situations at 5000 ad and 10 000 ad, and three different climates (normal and wet temperate climates and a cold climate with permafrost); the simulation cases with their P, T, and PET input data, and the results in terms of ET and R for each case are listed in Table S2 (in Electronic Supplementary Material). The simulations of future hydrology were carried out with and without taking landscape succession into account, which means that the regolith was either taken from the model for present conditions (also in the areas that changed from sea to land) or from the modeled future regolith distributions (Brydsten and Strömgren 2010).

The results in terms of water balances extracted for different times (shoreline positions), areas (present and future land and specific catchments), and climate conditions are also presented in Table S2 (in Electronic Supplementary Material), and indicate that the variations in the overall water balance are small as long as the climate is the same. The calculated distribution of the precipitation that falls during the year was approximately 30 % runoff and 70 % evapotranspiration for the normal temperate climate, more or less independently of the shoreline position and the regolith model considered. Differences related to the regolith model used were observed in the results, but these were not large enough to have significant effects on the overall water balances; for example, the variations in runoff among the cases studied were within ca. 10 %.

The other climate cases considered in the hydrological modeling were associated with changes in precipitation, temperature and other meteorological variables relative to the normal temperate case. The wet temperate case used an annual precipitation of ca. 1600 mm (almost three times the long-term average for present conditions). This case also included higher temperatures and much higher potential evapotranspiration, which led to comparatively small changes in the relative proportions of evapotranspiration (75 % of the precipitation) and runoff (25 %). However, there was a large absolute increase in the yearly runoff, 395 mm compared to 196 mm for the 10 000 ad model for temperate conditions, implying approximately a doubling of the runoff compared with the normal temperate case.

Some significant changes in the overall water balance appeared in the model results for a cold periglacial climate, where the climate conditions included a yearly precipitation of c. 400 mm. When applying a periglacial climate and permafrost conditions in the model [see Bosson et al. (2012a) for details on the model setup], the distribution of the precipitation was approximately 50 % runoff and 50 % evapotranspiration. This implies that in absolute terms the runoff is similar to that in the normal temperate case, whereas the evapotranspiration is much smaller in the case of a cold climate. An important consequence of the cold climate considered in this case is that the periglacial system contains a thick permafrost layer, which reduces the possible pathways for groundwater and significantly affects flow and hydrological interactions between the surface and the bedrock (see Bosson et al. 2012b).

**Fig. 4:**
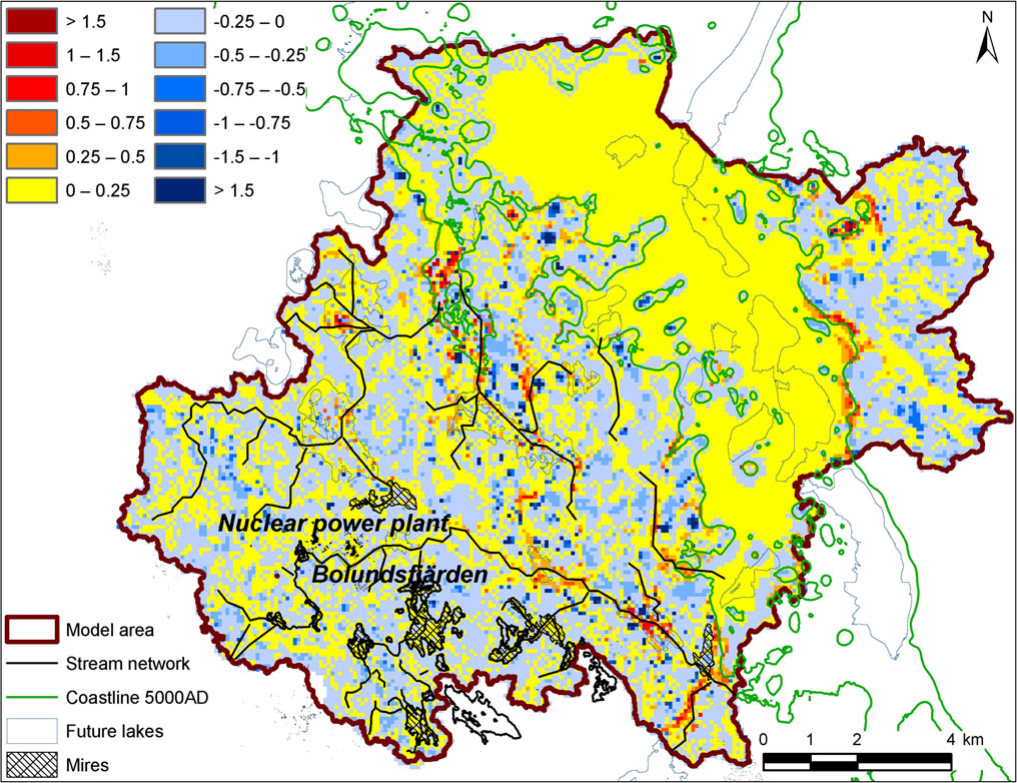
Discharge (*brown* to *yellow*) and recharge areas (*different shades of blue*) in the regolith, calculated using the MIKE SHE model with the 5000 ad shoreline and regolith distribution. Discharge and recharge conditions are defined by the head difference (in meters) between the two regolith layers in the model (the regolith is represented by two layers). The *continuous yellow area* is related to the presence of the sea; it represents the relatively small upward gradients below the sea. Modified from Bosson et al. (2010)

In Fig. 4, showing results from the 5000 ad simulation with normal temperate climate, the model area is divided into subareas indicating where groundwater recharge and discharge may take place. The different areas are identified using the calculated head difference between the two regolith layers in the model (i.e., the two uppermost layers), such that discharge areas are identified as areas with an upward hydraulic gradient and recharge areas are those with a downward gradient. The model results indicate that new lakes and streams, formed due to landscape succession, are discharge areas and the relatively higher-altitude areas are recharge areas, i.e., the same pattern as observed for present conditions at the site. The distribution of recharge and discharge areas changes somewhat with shoreline displacement, such that some areas with larger vertical gradients form in the new land areas (cf. the red areas in Fig. 4), but the overall pattern with mostly relatively small recharge and discharge areas in the terrestrial areas is the same for all shoreline locations and regolith models.

Individual objects such as specific lakes and wetlands obviously change significantly in response to landscape succession. For example, some of the presently existing lakes in Forsmark change into mires during the period from 2000 ad to 5000 ad, and are marked as mires in Fig. 4. However, since the terrestrialized lakes are still topographical low points, most of them remain as discharge areas as long as a temperate climate prevails at the site. There are some exceptions, but in general the overall groundwater flow pattern seems to be governed by the topography and climate, and not so much by the stratigraphy, thickness, or type of regolith.

The groundwater table is correlated to the topography also in the future models. However, comparing the depth to the groundwater table for present and future conditions in the area constituting land today (Fig. S3 in Electronic Supplementary Material) it is seen that the groundwater depths are in general larger in the models describing future site conditions. As the shoreline becomes more distant the groundwater levels drop. The cumulative frequencies of groundwater depth show that the groundwater table is deeper in the future models, but still 80 % of the model area has groundwater depth within 2 m of the ground surface at both 5000 ad and 10 000 ad.

## Discussion and Conclusions

The present conditions at the site are central when attempting to model future site hydrology. In particular, the present site serves as an initial state in the modeling of the future. Furthermore, by studying a range of hydrological objects of a certain type (e.g., lake-mire objects) representing different distances from the present coastline, different stages in the on-going succession of the objects can be observed and modeled. Historical records of the main processes driving site development, such as shoreline displacement, can be collected and used to construct descriptions of possible futures. The confidence in models describing the future relies to a large extent on the ability of the models to describe the present state and reproduce measured data and other observations from the site. Extensive model calibration and other comparisons between measurements and model results were performed as a part of the modeling presented herein, and the resulting numerical models showed good agreement with field data for realistic values of model parameters.

Shoreline displacement is a major process in the development of the (presently) coastal Forsmark site. It determines the overall distribution of land and sea, and when a certain area emerges from the sea and hence when lakes and streams are formed. The change from sea to land also has a significant influence on the groundwater flow pattern. The results presented in this paper emphasize the importance of the climate relative to other factors for general hydrological features such as water balances. Clearly, to further investigate the sensitivity of site hydrology to meteorological variables is an important topic for additional studies. Other processes affecting the hydrology, primarily processes related to lake succession and terrestrialization, are obviously of large significance when performing more detailed studies, especially of the future development of individual hydrological objects.

Changes in the climate may have more profound effects on flow systems than just to change magnitudes of flow components. In particular, for a cold climate Bosson et al. (2012b) showed that many discharge areas for deep groundwater could turn into recharge areas when permafrost (i.e., frozen ground during more than 1 year) is present. This is an effect of the freezing of the ground and the formation of ‘taliks’ that determine where groundwater flow takes place. A talik is an unfrozen ‘window’ in the otherwise frozen permafrost layer, which may form under larger surface water bodies and through which groundwater may flow between the surface (seasonally unfrozen groundwater systems in the regolith or the lake itself) and the unfrozen bedrock below the permafrost layer. The taliks form in areas that are discharge areas under temperate conditions, and since they are the only passages through the permafrost in the periglacial system they are the only places where recharge can take place under such conditions. Detailed evaluations comparing present hydrological conditions at Forsmark with a possible future periglacial system are presented by Bosson et al. (2012a, b).

The calculations of hydrological fluxes for biosphere objects (see Appendix, Electronic Supplementary Material) represent the most detailed model scale considered in the present work. However, these results are extracted from larger-scale, distributed hydrological models, in this case MIKE SHE models covering many potential biosphere objects. An obvious next step from the present use of an ‘average object’ is to perform flow calculations for the specific biosphere objects included in the radionuclide transport model, and then use the results in an object-specific parameterization in the transport model. The hydrological models are also used to support the subdivision of biosphere objects into compartments; this idealization of the system should be revisited when developing new models. Furthermore, the possibilities of providing fully dynamic flow modeling results, i.e., hydrological fluxes that change during the succession from lake to mire and further, should be investigated.

## Electronic supplementary material

Below is the link to the electronic supplementary material.

Supplementary material 1 (PDF 861 kb)
