# Supplementary material for: From Site Data to Safety Assessment: Analysis of Present and Future Hydrological Conditions at a Coastal Site in Sweden
Source: Ambio. 2013 Apr 26;42(4):425–34. doi: 10.1007/s13280-013-0394-6 (PMC3636372; doi:10.1007/s13280-013-0394-6)
Supplement: Supplementary file 1 — Supplementary material 1 (PDF 861 kb) [file 13280_2013_394_MOESM1_ESM.pdf]

Electronic Supplementary Material

From Site Data to Safety Assessment: Analysis of Present and Future Hydrological Conditions at a Coastal Site in Sweden

Sten Berglund, Emma Bosson, Mona Sassner

GENERATION OF INPUT DATA TO RADIONUCLIDE MODELING

In order to calculate the water fluxes to and within biosphere objects using the hydrogeological model, detailed water balances were extracted from the MIKE SHE water flow simulations (Bosson et al. 2010). The water balance output included fluxes between the model area and its surroundings, internal fluxes between model compartments, storage changes, and measures of errors in the results (i.e., water balance errors due to the numerical calculations as such). In MIKE SHE, water balances can be generated at a variety of spatial and temporal scales and in a number of different formats. In this case, separate water balances for the lake and mire parts and integrated balances for the lake and mire areas were extracted (see Bosson et al. [2010] for details).

Six of the present lake and mire areas in Forsmark were selected for supplying the water balance data (Figure S1). Existing lakes in their present states were selected for this modeling (instead of modeled future ones), because these are the objects for which the most extensive data are available. This implies that the present lakes are assumed representative of both present and future biosphere objects. As described in connection with the model results below, the selected objects include a large variation in the sizes of objects and their relative proportions of lake and mire areas. For each selected object, the water balances were calculated for a one-year period, and the results were taken to represent annual mean values. The radionuclide modeling considered one representative object, which was parameterized by calculating the arithmetic mean of each flow component using the results from the six objects.

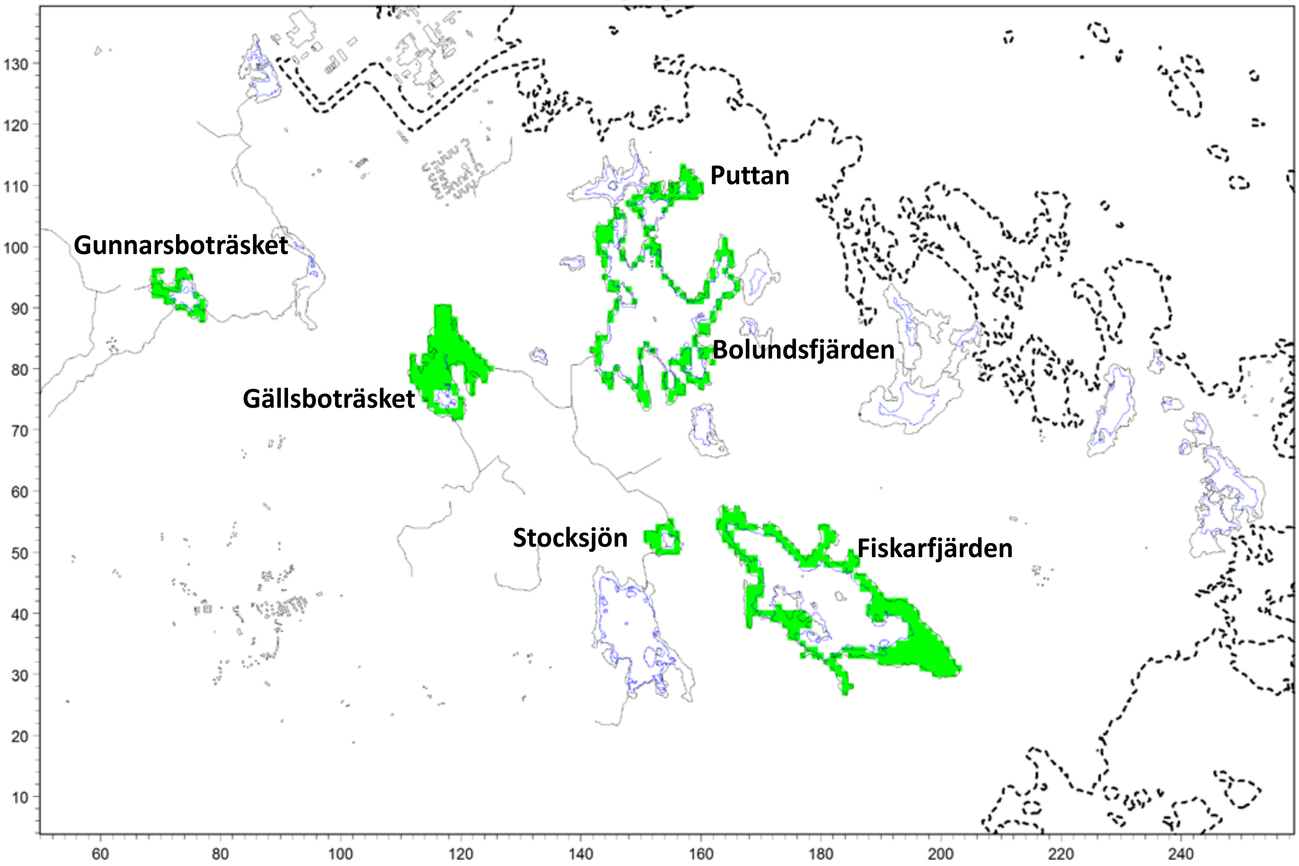

Fig. S1 The six lake and mire areas that were selected for extraction of water-balance data to be used in the biosphere modeling of radionuclide transport. The green areas represent the mires surrounding the lakes and the dotted line is the present shoreline. The length scale of the map is expressed in number of grid cells, which in this case means that distances in meters are obtained through multiplication by 40. Reproduced from Bosson et al. (2010)

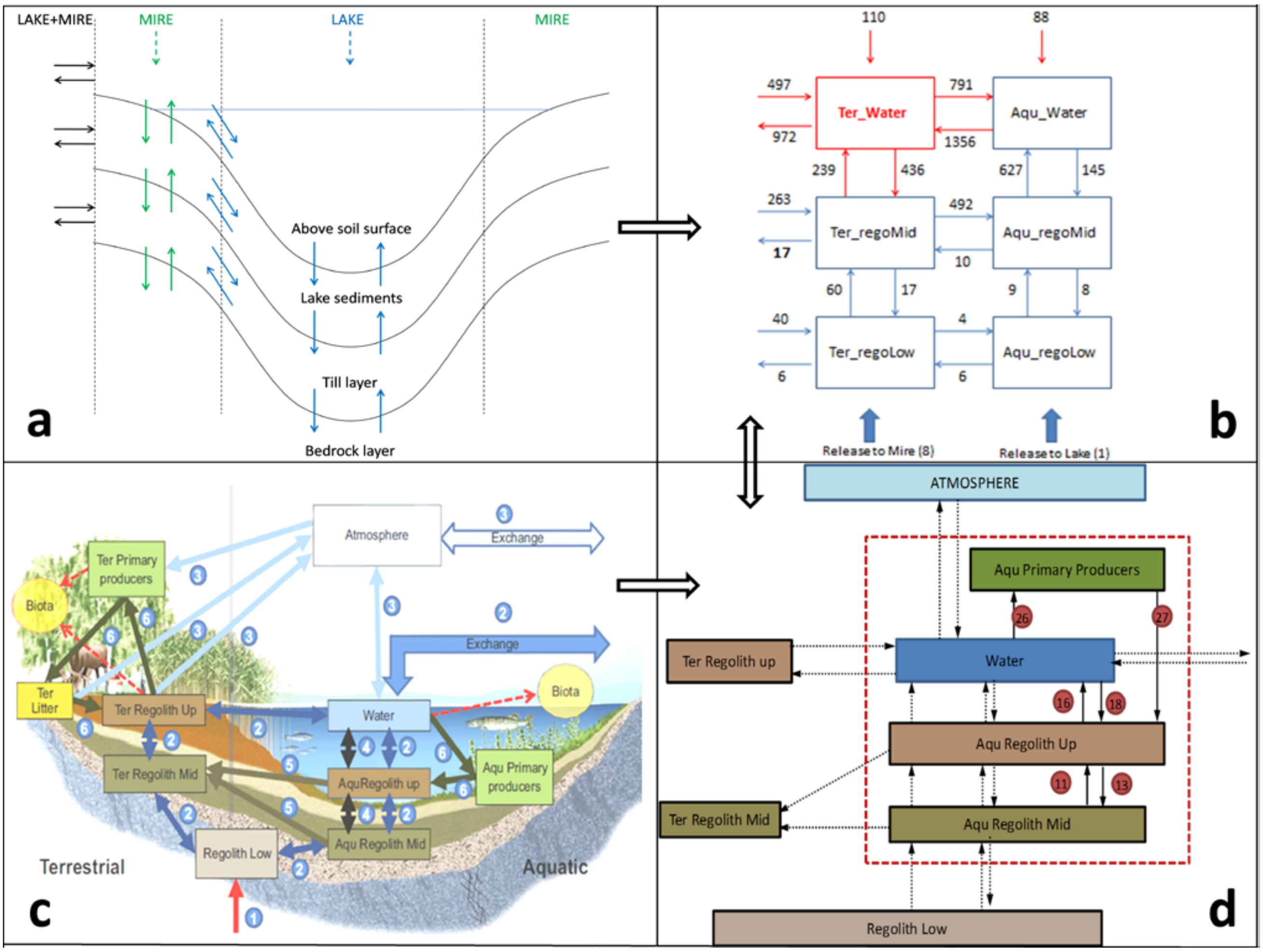

Fig. S2 Illustration of hydrological and radionuclide transport models, and how data are transformed in the parameterization of the latter: (**a**) schematic section of lake-mire area in the hydrological (MIKE SHE) model, (**b**) compartment representation of averaged hydrological fluxes, (**c**) conceptual model for radionuclide transport, and (**d**) compartment model implemented in the biosphere radionuclide transport calculations. In (b), (c), and (d) 'Ter' refers to the mire part of the object and 'Aqu' to the lake part; 'Regolith Low' (or 'regoLow') is the till layer, whereas 'Regolith Mid' and 'Regolith Up' consist of clays and various other regolith materials overlying the till (including lake sediments), see Löfgren (2010) for details

Figure S2 illustrates the handling of the hydrological modeling results, i.e., how water balances were extracted, and the interactions between the hydrological model and the conceptual and numerical models used to describe radionuclide transport. Figure S2a, the upper left figure, shows a schematic cross-section of a lake and mire area, including the external and internal boundaries and the calculation layers in the MIKE SHE model. Since the objects are not delimited by water divides (they are defined based on the maximum historical extent of the lake), each object-specific water balance contains horizontal fluxes across the object boundary, in addition to the various internal fluxes between calculation layers, lake and mire areas, surface water, and groundwater. The water exchange between the till layer and the underlying bedrock is an internal exchange in the MIKE SHE model and an exchange across the model boundary in the radionuclide model.

Figure S2b shows an idealized description of the average fluxes calculated in the MIKE SHE model, where the boxes represent the different parts of the MIKE SHE section in Figure S2a. Extraction of water balances for individual objects and averaging among the objects resulted in the fluxes indicated in the figure; however, the results are not discussed here. The lower left figure, Figure S2c, shows the conceptual model of the processes included in the radionuclide transport model, with arrows indicating fluxes between different parts of the ecosystem (Avila et al. 2010); note that not all fluxes are transport by water. In the conceptual model (c), the

radionuclide input from the bedrock is represented by a red arrow (1), whereas processes of importance for radionuclide transport are indicated as follows: dark blue arrows indicate water fluxes (2), light blue arrows gas fluxes (3), black arrows sedimentation/resuspension fluxes (4), brown arrows wetland growth (5), dark green arrows biological uptake/decomposition (6), and arrows marked 'exchange' export to and import from the surrounding landscape.

This conceptual model was developed into the compartment model illustrated in Figure S2d, which was used in the radionuclide transport modeling described by Avila et al. (2010). The compartments in this transport-based description were not identical to those in the hydrologically based idealization of the system (Fig. S2b); some adjustments and simplifications were necessary to comply with the overall structure and needs of the transport model. For instance, the fluxes had to be expressed in terms of parameters that could be scaled to the sizes of biosphere objects and their catchment areas, in order to enable the use of object-specific geometrical information in the transport model. A detailed description of the transformation from the compartment representation of the MIKE SHE model to the compartment model used in the radionuclide modeling is presented by Löfgren (2010).

RESULTING HYDROLOGICAL FLUXES

Applying the methodology illustrated in Figure S2, external and internal water fluxes for the present temperate climate were calculated for lake-mire objects shown in Figure S1 and used to parameterize the biosphere objects in the radionuclide transport and radiation dose model with averaged water fluxes from the hydrological modeling. The succession of a biosphere object was taken into account by increasing the proportion of the terrestrial part of the object during the simulation (Avila et al. 2010). A detailed description of the fluxes delivered to the radionuclide model and how they were transformed and used there is outside the scope of the present paper (see Löfgren [2010] and Avila et al. [2010] for details). However, a summary of the results pertaining to the MIKE SHE compartments shown in Figure S2b is presented below.

Table S1 shows geometrical data and modeling results in terms of water fluxes to and from selected lake-mire objects. In particular, the results in the table include the 'mean object' delivered to and used in the radionuclide modeling and three of the six objects that were considered in the hydrological modeling (Fig. S1). As indicated by the geometrical data, the selected objects represent relatively large variations in sizes and lake fractions. They can be categorized as large objects with large lake areas (represented by Lake Bolundsfjärden), intermediate size with a small lake (Lake Gällsboträsket) or small with an intermediate lake fraction (exemplified by Lake Gunnarsboträsket). The mean object has an area of c. 0.3 km^2 of which almost half is lake.

All flow components in Table S1 are normalized with the total amount of water entering the area, which in this case is calculated as the sum of the net precipitation (total precipitation minus total evapotranspiration), the external horizontal inflows, and the vertical flow from the bedrock to the regolith. The largest inflow is on the surface for most of the lakes; this contribution is on average approximately 50%. The surface inflow is the surface water flow reaching the boundary of the studied area coming from the upstream parts of the catchment area. However, also the inflow through the sediment layers is large (26% for the mean object), and for the small objects it constitutes the largest inflow. The net precipitation is on average c. 20% of the total inflow, whereas the inflow from the bedrock is very small, on the order of 1%.

The outflow is dominated by outflow on the surface, which represents the runoff to and further with the stream that goes through each lake and mire area. A closer inspection of the results for the internal fluxes (Fig. S2b) reveals that there are relatively large net fluxes from the mire to below the lake in the sediment layers, and then up to the lake water and further from the lake to surface water outside it. These fluxes represent the various groundwater and surface water flows that take place near the lake shoreline. As described by Bosson et al. (2008), this part of the modeled domain is very active and complex from a hydrological point of view, with interactions between surface water and groundwater, groundwater inflow from the surroundings, and flow from and to sediment layers below the lake. The calculated internal fluxes are much larger in the upper part of the model than at larger depths in the regolith.

Table S1 Geometrical data and results of flow modeling for three of the six objects considered in the generation of input to the radionuclide modeling (Fig. S1) and for the mean object for which data were delivered (Fig. S2b). Flow 'on surface' refers to surface water in streams or overland water on the ground surface, and 'sediments' to the upper layers in the regolith, consisting of different types of regolith materials, that overlie the till layer residing on the bedrock

	**Mean object**[1]	**Lake Bolunds-fjärden**	**Lake Gällsbo-träsket**	**Lake Gunnarsbo-träsket**
Area of object (lake+mire, km^2)	0.30	0.62	0.21	0.07
Lake fraction (%)	47.5	62.9	9.5	28.6
**Inflow (% of total inflow)**				
Net precipitation	19.7	24.0	9.2	8.4
Horizontal inflow on surface	49.3	49.1	71.7	33.3
Horizontal inflow to sediments	26.1	19.6	17.0	56.2
Horizontal inflow to till layer	4.0	6.2	1.5	1.2
Vertical inflow from bedrock	0.9	1.1	0.6	0.9
**Outflow (% of total inflow)**				
Horizontal outflow on surface	96.5	98.2	97.0	97.7
Horizontal outflow from sediments	1.7	0.6	2.5	0.1
Horizontal outflow from till layer	0.6	0.1	0.3	0.1

[1]Arithmetic average of six objects

Table S2 Climate input data and calculated annual water balance for each simulation case. The upper part of the table lists the results for the temperate simulation cases for the area constituting land at present in Forsmark (2000 AD) and the lower part lists the results for the area constituting land at each time studied (as given by the future shorelines, Fig. 3 in the main [printed] document). Temperature (T), potential evapotranspiration (PET), and precipitation (P) are input data, whereas total evapotranspiration (ET) and total runoff (R) are results of the simulations. Note that the modeling is transient and that input data are given as time series of daily values; the table presents annual mean (T) and sums (PET and P) of these daily values

**Annual water balance results for the present land area**							
	**Climate conditions**			**Absolute and relative hydrological flows**			
**Case**	**T (°C)**	**PET (mm/y)**	**P (mm/y)**	**ET (mm/y)**	**R (mm/y)**	**ET/P (-)**	**R/P (-)**
Temperate, 2000 AD	+6.4	420	583	405	175	0.69	0.30
Temperate, 5000 AD	+6.4	420	583	410	188	0.70	0.32
Temperate, 10 000 AD	+6.4	420	583	400	196	0.69	0.32
**Annual water balance results for the area constituting land at each time studied (2000 AD, 5000 AD or 10 000 AD)**							
	**Climate conditions**			**Absolute and relative hydrological flows**			
**Case**	**T (°C)**	**PET (mm/y)**	**P (mm/y)**	**ET (mm/y)**	**R (mm/y)**	**ET/P (-)**	**R/P (-)**
Temperate, 2000 AD	+6.4	420	583	405	175	0.69	0.30
Temperate, 5000 AD	+6.4	420	583	408	185	0.70	0.32
Temperate, 10 000 AD	+6.4	420	583	403	186	0.69	0.32
Periglacial, 10 000 AD	-7.0	216	412	193	217	0.47	0.53
Wet, 10 000 AD	+7.7	2062	1568	1190	395	0.76	0.25

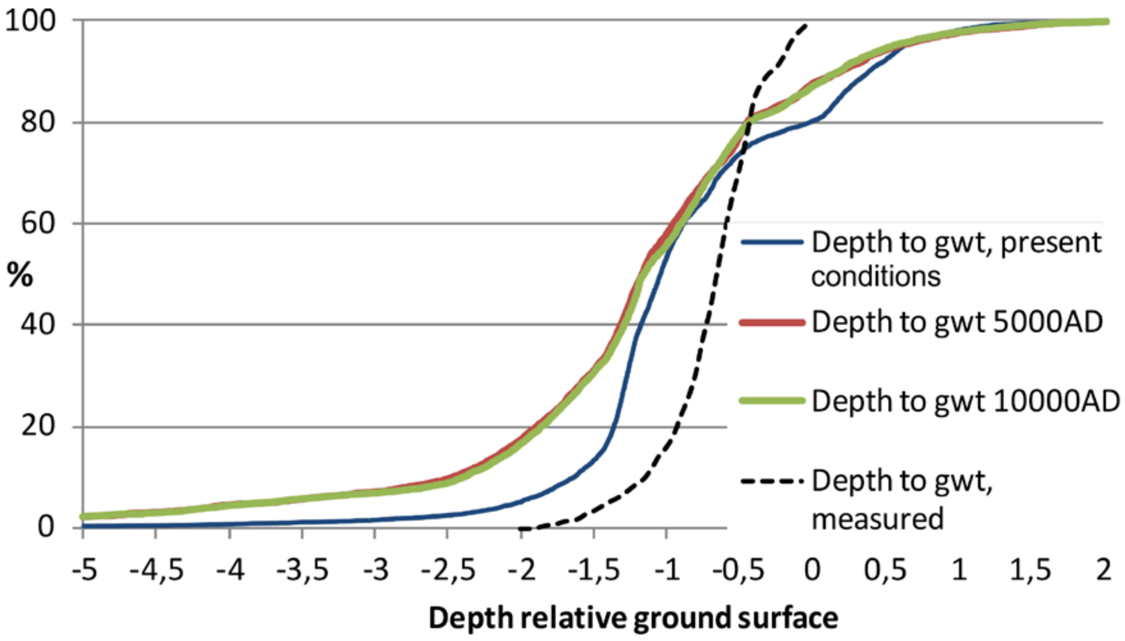

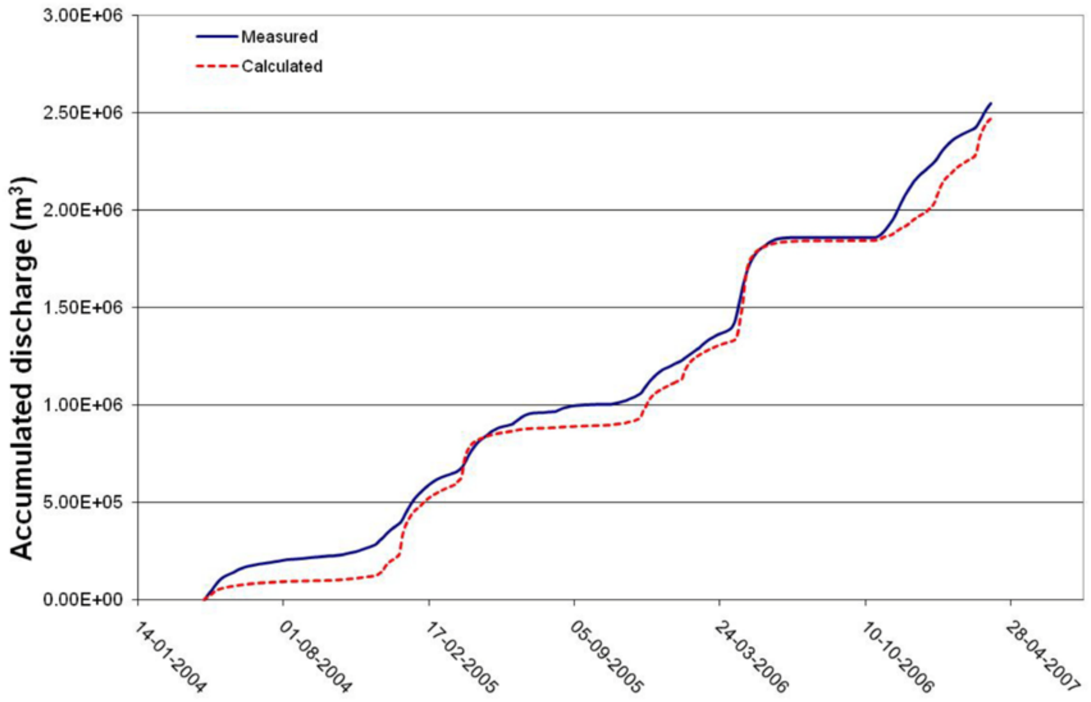

Fig. S3 Examples of results from the numerical modeling and comparisons with data from field measurements. The upper figure shows cumulative groundwater depth frequencies (groundwater table depths relative to ground surface) from models representing different times and from measurements in the groundwater monitoring wells; the model results represent the whole land area at each time, whereas the measured depths are for the monitoring well locations only. The bottom figure shows measured and calculated accumulated surface water discharges at one of the stations in the Forsmark area. Comparisons of this type were used in the model calibration

REFERENCES

Avila, R., P.-A. Ekström, and P.-G. Åstrand. 2010. Landscape dose conversion factors used in the safety assessment SR-Site. Svensk Kärnbränslehantering AB, SKB TR-10-06, Stockholm, Sweden, Report, 169 pp.

Bosson, E., L.-G. Gustafsson, and M. Sassner. 2008. Numerical modelling of surface hydrology and near-surface hydrogeology at Forsmark. Site descriptive modelling, SDM-Site Forsmark. Svensk Kärnbränslehantering AB, SKB R-08-09, Stockholm, Sweden, Report, 246 pp.

Bosson, E., M. Sassner, U. Sabel, and L.-G. Gustafsson. 2010. Modelling of present and future hydrology and solute transport at Forsmark. SR-Site Biosphere. Svensk Kärnbränslehantering AB, SKB R-10-02, Stockholm, Sweden, Report, 366 pp.

Löfgren, A. 2010. The terrestrial ecosystems at Forsmark and Laxemar-Simpevarp. SR-Site Biosphere. Svensk Kärnbränslehantering AB, SKB TR-10-01, Stockholm, Sweden, Report, 444 pp.
